# Melatonin Ameliorates Corticosterone-Mediated Oxidative Stress-Induced Colitis in Sleep-Deprived Mice Involving Gut Microbiota

**DOI:** 10.1155/2021/9981480

**Published:** 2021-06-23

**Authors:** Ting Gao, Zixu Wang, Jing Cao, Yulan Dong, Yaoxing Chen

**Affiliations:** College of Veterinary Medicine, China Agricultural University, Haidian, Beijing 100193, China

## Abstract

**Background:**

Inflammatory bowel disease (IBD) is a result of a complex interplay, making development of a specific treatment a challenging task. Corticosterone was considered a risk factor of stress relative enteritis. Our previous studies found that melatonin exerts an improvement effect in sleep deprivation (SD)- induced corticosterone overproduction and colitis. A present study further explored the mechanism whereby melatonin prevented corticosterone-mediated SD-induced colitis.

**Methods:**

A 72-hour SD mouse model with or without melatonin supplementation and fecal microbiota transplantation (FMT) to investigate the core role of corticosterone in melatonin-mediated gut microbiota improving SD-induced colitis. Further, corticosterone-treated mice were assessed to the effect of melatonin on corticosterone-mediated gut microbiota dysbiosis-induced colitis. Meanwhile, an in vitro test studied modulatory mechanism of metabolite melatonin.

**Results:**

SD caused an excessive corticosterone, gut microbiota disorder and colitis phenotype. Similarly, corticosterone-supplemented mice also exhibited gut microbiota dysbiosis and colitis, and the FMT from SD-mice to normal mice could restore the SD-like colitis, but no change in the corticosterone level, which suggested that corticosterone-mediated intestinal microbiota imbalance plays a central role in SD-induced colitis. Further, we demonstrated melatonin-mediated MT2 weakened GR feedback, suppressed oxidative stress, restored the intestinal microbiota and its metabolites homeostasis, and inactivated the STAT3/AP-1/NF-*κ*B pathway-induced inflammatory response in vivo and in vitro.

**Conclusions:**

We revealed that excessive corticosterone is a core risk factor for SD-induced colitis and provided a better understanding of the effects of melatonin, expected to be a personalized targeted therapy drug, on corticosterone-mediated gut microbiota inducing colitis.

## 1. Introduction

Modern lifestyle, which includes long working hours and commuting times, psychological stress, personal choices, and social and family demands, has led to an increase in short sleep durations [1]. Acute periods of sleep deprivation (SD) can result in intestinal barrier dysfunction, including intestinal mucosal damage, and intestinal microbiota disturbance in rodents [2], which can be a risk factor for frequent inflammatory bowel disease (IBD) [3, 4]. IBD pathogenesis has been linked to the presence of symbiotic microorganisms living in the intestinal tract [5, 6]. Research has also suggested that IBD-associated genetic defects can lead to pathobiont accumulation and penetration into the intestinal tissue, which further promotes dysbiosis and inflammation [6]. Sleep plays an integral role in intestinal health and also has been shown to significantly impact intestinal microbiome and metabolism [2, 7]. However, whether SD-mediated intestinal microbiota disorders induced intestinal mucosal damage and eventually lead to the occurrence of IBD has yet to be investigated.

Previous researches demonstrated that SD, which may be a stressor, could activate the hypothalamic-pituitary-adrenal (HPA) axis in rats [8], which triggered the synthesis and production of corticosterone (CORT) in a dynamic manner [9]. Recent studies have shown that CORT may cause an imbalance in the gut microbes [10, 11], including the downregulation of intestinal microbiota richness and relative abundance of Bacteroidetes and an increase in Firmicutes and Proteobacterium. Considering that the gut microbiota constitutes the intestinal barrier, promotes the continuous existence of the gut microbiota, stimulates intestinal epithelial cell regeneration, produces mucus, and nourishes mucosa [12], we assessed the impact of SD-induced IBD related to the increase of CORT production-mediated intestinal microbiota dysbiosis.

Melatonin (MT) is a circulating hormone primarily produced from tryptophan by the pineal gland. This hormone has received widespread interest because it acts as a homeostatic regulator of the HPA axis [13], which is associated with diminished overall CORT secretion and increased sensitivity to CORT feedback. Moreover, as a molecule with many functions that serves as the primary signal mediating microbial metabolism, circadian rhythms, and intestinal mucosal immune cells, MT has potential for use in treating intestinal diseases [14]. However, whether MT shapes intestinal biological functions through the CORT-mediated intestinal microbiota has yet to be investigated.

Therefore, in the current study, we (1) use mouse faeces microbiota transplant (FMT) experiment to investigate SD-mediated intestinal microbiota disorder-induced colitis and to investigate whether MT administration could improve SD-induced colitis via restoring gut microbiota homeostasis; (2) further verify the core role of excessive CORT-mediated intestinal microbiota imbalance in SD-induced colitis and verify whether MT administration could suppress this process; and (3) explore the signaling pathway in MT improving CORT-mediated intestinal microbiota disorder and intestinal mucosal injury using in vivo and in vitro.

## 2. Materials and Methods

All experiments were conducted according to the Guide for the Care and Use of Laboratory Animals published by the Animal Welfare Committee of the Agricultural Research Organization, China Agricultural University (Approval No. CAU20170911-2).

### 2.1. Animals and Treatments

A total of SPF 132 male ICR mice (8 weeks old; Vital River Laboratory Animal Technology Co. Ltd., Beijing, China) were housed in 22 cages (6 mice/cage) under conventional conditions. After acclimatisation for one week, the mice were randomly divided into eleven groups: sleep deprivation (SD), SD+MT supplementation (SD+MT), only MT supplementation (MT), non-sleep-deprived control (CON) groups, fecal microbiota transplantation (FMT) from mice of the CON group (F-CON), FMT from mice of the SD group (F-SD), FMT from mice of SD+MT group (F-SM), FMT from mice of vehicle (F-R), only corticosterone supplementation (CORT), CORT+MT supplementation (CORT+MT), and non-CORT-treated vehicle group (CON). Specific operation is provided in the Supplementary Material.

### 2.2. Faecal Material Preparation and FMT Regime

Faecal material was collected from mice of the CON, SD and SD+MT groups in SD experiment and placed into Eppendorf tubes containing freezing solution (sterile saline solution with 12.5% glycerol) and homogenized [15]. For more details, see the Supplementary Material.

### 2.3. Cell Culture and Treatment

Mouse primary colonic intestinal epithelial cells (IECs, BALB-5047, USA) were cultured in 96-well plates (5 × 10^6^ cells/mL) and 12-well plates (5 × 10^5^ cells/mL). Some CORT-treated IECs (10 *μ*M, Solarbio Ltd., Beijing, China; CORT-cells) were treated with 100 *μ*M NAC (a free radical scavenger; MCE, New Jersey, USA; CORT+NAC-cells), 2 *μ*M MT (Sigma-Aldrich, St. Louis, USA; CORT+MT-cells) or 20 *μ*M RU-486 (a selective GR antagonist; MCE, New Jersey, USA; CORT+RU-486-cells). After MT supplementation for 30 min, some CORT+MT-cells were sequentially treated with 10 mM 4P-PDOT (a nonselective MT2 antagonist; MCE, New Jersey, USA; CORT+MT+4P-PDOT-cells). Each plate of treated cells was incubated for 24 h.

The cells from the 96-well plates were assessed for proliferation activity using 3-(4,5-dimethylthiazol-2-yl)-2,5-diphenyltetrazolium bromide (MTT, Sigma-Aldrich, St. Louis, MO, USA) (optical density was determined using a microplate reader (Model 680, Bio-Rad, St. Louis, MO, USA) equipped with a 570 nm wavelength filter) and reactive oxygen species (ROS) (Nanjingjianchen, Beijing, China) assays. A ROS assay kit was purchased from Sigma-Aldrich and used according to the manufacturer's instructions (*n* = 9). The intracellular ROS generation was measured using a flow cytometer with an oxidation-sensitive DCFH-DA fluorescent probe. The suspension was loaded using DCFH-DA solution at a final concentration of 50 M and was incubated for 30 min at 37°C. Then, the samples were centrifuged at 1,000 rpm for 5 min (4°C), and the cells were resuspended in phosphate-buffered saline (PBS, pH 7.2-7.4). For each treatment, 19105 cells were counted, and the experiment was performed in triplicate. Fluorescence was detected using a fluorescence microplate reader (excitation 488 nm and emission 525 nm) [16]. The cells from the 12-well plates were collected for western blotting. Each assay used a repeat of 8 wells.

### 2.4. Enzyme-Linked Immunosorbent Assay (ELISA)

Plasma samples were collected for the detection of CORT; colon samples were collected for the detection of inflammatory factor (TNF-*α*, IL-10 and IL-17) concentrations using a competitive ELISA assay (USCN Life Science, Inc., Wuhan, China). All tests were performed according to the manufacturer's instructions. Eight samples were used in each group. Each sample was tested in triplicate. The data are expressed as ng/mL for plasma CORT levels and pg/mg protein for TNF-*α*, IL-10, and IL-17 levels in the colon tissue.

### 2.5. Intestinal Permeability to Fluorescein Isothiocyanate (FITC)- Dextran

Before the experiment ended at 6:00 am, all mice were deprived of food for 2 h and orally gavaged with 0.6 mg/g body weight of 4 kDa FITC-dextran at a concentration of 80 mg/mL 1 h before euthanasia. The FITC fluorescence in the serum was measured using a fluorescence spectrophotometer with wavelengths of 485 nm (excitation) and 535 nm (emission) [17]. A standard curve was created by diluting FITC-dextran in PBS. The concentration of FITC-dextran in the serum was calculated using the standard curve.

### 2.6. Immunohistochemical Staining

We used immunohistochemistry to stain for MUC2, ZO-1, and Claudin-1 in paraffin intestinal sections. Sections were incubated overnight at 4°C with the monoclonal rabbit anti-mouse primary antibody (MUC2, 1 : 500; ZO-1, 1 : 200; Claudin-1, 1 : 200; Abcam, Cambridge, MA, USA). Specific operation is provided in the Supplementary Material.

### 2.7. Western Blotting

Portions of the colonic segments and IEC samples (*n* = 9) were rapidly homogenized in liquid nitrogen and stored at -80°C for western blotting analysis. For more details, see the Supplementary Material.

### 2.8. Colonic RNA and Fecal DNA Isolation and Quantitative RT-PCR Analysis

Colonic RNA was isolated using the RNeasy Mini Kit (Qiagen, Germantown, MD, USA) following the manufacturer's instructions. The quantity and integrity of the RNA were assessed spectrophotometrically with a Nanodrop apparatus (Thermo Fisher Scientific). Fecal DNA was isolated using the microflora DNA extraction kit (MoBio, 12988-10, USA) following the manufacturer's instructions. Specific DNA sequences were amplified with a Bio-Rad CFX Connect real-time PCR device (Hercules, CA, USA). The primers used are shown in Table 1.

### 2.9. Microbial Sequencing

The 16S rRNA gene was amplified using PCR and composite specific bacterial primers as described previously [2]. For more details, see the Supplementary Material.

### 2.10. Metabolomics Profiling

We performed LC-MS analyses on a quadrupole-time-of-flight (Q-TOF) 6510 mass spectrometer (Agilent Technologies, Santa Clara, CA, USA) with an electrospray ionization source [18]. For more details, see the Supplementary Material.

### 2.11. Statistical Analysis of Data

Data are expressed as the mean ± standard error and were analyzed using SPSS 10.0 statistical software (SPSS, Inc., Chicago, IL, USA). Differences between groups were statistically analyzed using ANOVA followed by two-way ANOVA, which was used to determine the significance of differences among groups (*p* < 0.05 and *p* < 0.01).

## 3. Results

### 3.1. Effect of MT Supplementation on SD Enhances CORT Secretion and Intestinal Microbiota Disorder in the Colon

To clarify whether MT improved SD-induced CORT overproduction and intestinal dysbiosis, we established an acute continuous 72 h SD mouse model. The results showed a significant increase in the ROS content (47.6%, *p* = 0.004; Figure S1A), expression level of GR protein (52.6%, *p* = 0.001; Figure S1B), and HSP90 mRNA (50.9%, *p* ≤ 0.001; Figure S1C), as well as a decrease in the HSP70 (38.1%, *p* = 0.004; Figure S1D) and P23 mRNA (25.6%, *p* = 0.014; Figure S1E) in the SD group compared with that in the CON group. However, MT pretreatment effectively reversed these SD-induced changes. Next, analysis of intestinal microbiota composition was performed (Figure S1F-I), and results showed an upregulation of relative abundance in Firmicutes (51.9%, *p* ≤ 0.001, Figure S1G) and Proteobacterium (67.3%, *p* = 0.009, Figure S1H) and a downregulation of Bacteroidetes (15.7%, *p* = 0.042, Figure S1F) and Prevotellaceae (30.1%, *p* = 0.016, Figure S1I) in the SD group compared with that in the control group. By contrast, MT pretreatment attenuated these effects of SD on the intestinal microbiota disorder. Consequently, MT pretreatment attenuated the excessive CORT and intestinal microbiota imbalance in SD mice.

### 3.2. FMT Promotes Reestablishment of the Colitis Phenotype and Microbiota Disorder in Mice

We explore whether FMT from the SD mice induced a colitis phenotype in receiving mice. The present results demonstrated that there was a remarkable reduction in the expression level of MUC2, TJ (ZO-1 and Claudin-1) and Card9 proteins by 43.2% (*p* = 0.001, Figures S2A and D), 63.9% (*p* ≤ 0.001, Figures S2B and E), 35.6% (*p* = 0.021, Figures S2C and F), and 29.8% (*p* = 0.034, Figure S2I), as well as a significant increase in the DAI score, intestinal permeability, and colonic IL-17 level by 36.1% (*p* = 0.003, Figure S2G), 42.6% (*p* ≤ 0.001, Figure S2H), and 78.3% (*p* ≤ 0.001, Figure S2J) in the F-SD group compared with that in the F-CON group. However, the stimulatory effects of F-SD on changes in colitis were reversed in the colon by F-SM supplementation.

Further, we examined whether F-SD caused an intestinal microbiota disorder. The relative abundance of Firmicutes and Proteobacterium was significantly elevated by 36.3% (*p* = 0.010, Figure S2L) and 57.7% (*p* ≤ 0.001, Figure S2N) in the F-SD group, respectively, versus the F-CON group. By contrast, Bacteroidetes and Prevotellaceae contents were significantly reduced by 32.5% (*p* = 0.032, Figure S2K) and 46.3% (*p* = 0.010, Figure S2M) in the colon of the F-SD group versus that of the control group. However, in the F-SM and F-R groups, all index was restored to the level of the F-CON group, resulting in no statistically significant differences among the F-SM, F-R and F-CON groups (*p* > 0.783).

In conclusion, SD-mediated intestinal microbiota imbalance induced colitis, while MT supplementation improved colitis via restoring intestinal microbiota dysbiosis.

### 3.3. Effect of MT Supplementation on CORT Treatment Impairs the Intestinal Mucosa Barrier, Mitochondrial Function, and Antioxidant Capacity of the Colon

**Figure 1:**
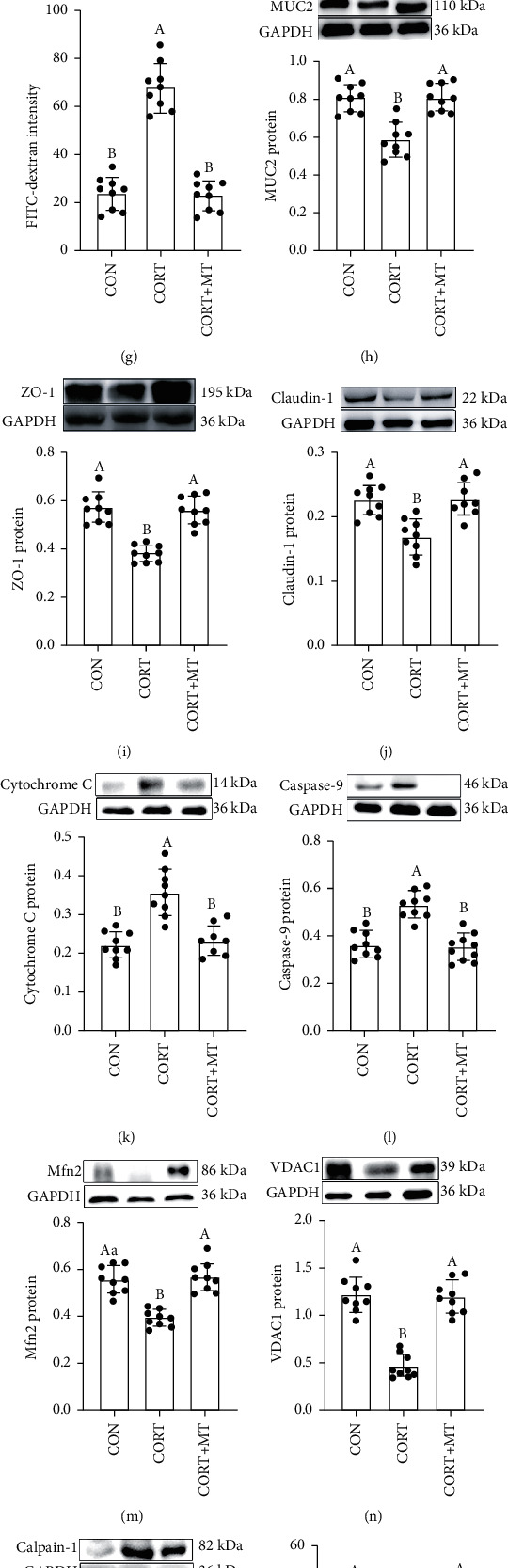
Effect of melatonin supplementation on improving CORT-induced intestinal barrier, mitochondrial function and antioxidant capacity impaired in mice. Serum CORT (a) concentrations; colonic length (b, c); H&E staining photographs (scale: 50 *μ*m) (d); histological score (e); DAI score (f); relative luciferase activity (g); colonic MUC2 (h), ZO-1 (i), Claudin-1 (j), Cytochrome C (k), Caspase-9 (l), Mfn2 (m), VDAC1 (n), and Calpain-1 (o) proteins; CAT (p), GSH-Px (q), SOD (r), T-AOC (s), and MDA (t) in the CON, CORT and CORT+MT groups. Values are presented as the mean ± SE. Differences were assessed using ANOVA and are denoted as follows: different lowercase letters: *p* < 0.05; different uppercase letters: *p* < 0.01; the same letter: *p* > 0.05.

Further, we observed the plasma CORT level significantly increased by 74.3% (*p* = 0.002, Figure 1(a)) in the CORT group compared with the CON group. Consistent with the increase in CORT, there was an increase in clinical score (202.9%, *p* = 0.003, Figures 1(a) and 1(e)), DAI score (69.9%, *p* ≤ 0.001, Figure 1(f)), intestinal permeability (67.8%, *p* = 0.003, Figure 1(g)), and a reduction in the colonic length (21.2%, *p* = 0.023, Figures 1(b) and 1(c)) and the levels of the MUC2 (27.8%, *p* = 0.010, Figure 1(h)), ZO-1 (39.4%, *p* = 0.007, Figure 1(i)), and Claudin-1 (47.7%, *p* = 0.030, Figure 1(j)) proteins in CORT-treated mice. Additionally, CORT caused increases in the expression levels of the Cytochrome C (160.3%, *p* = 0.029, Figure 1(k)) and Caspase-9 (51.9%, *p* ≤ 0.001, Figure 1(l)) proteins and reductions in the Mfn2, VDAC1, and Calpain-1 proteins by 30.1% (*p* = 0.006, Figure 1(m)), 15.7% (*p* = 0.002, Figure 1(n)) and (213%, *p* = 0.029, Figure 1(o)), respectively, compared with the CON group. Meanwhile, there was a decrease in the CAT (46.7%, *p* ≤ 0.001, Figure 1(p)), GSH-Px (15.7%, *p* = 0.003, Figure 1(q)), SOD (33.9%, *p* ≤ 0.001, Figure 1(r)) and T-AOC levels (44.5%, *p* = 0.001, Figure 1(s)) and an increase in the MDA level (29.7%, *p* = 0.010, Figure 1(t)) in CORT mice relative to the CON group. In contrast, MT supplementation attenuated the effects of CORT on the colonic dysfunction. The plasma CORT level, clinical score, DAI score, intestinal permeability, and expression levels of Cytochrome C, Caspase-9, and Calpain-1 proteins and MDA were decreased by 23.1-63.6% (*p* = 0.004-0.042), while the length of colon, expression levels of the TJ (ZO-1 and Claudin-1), Mfn2, VDAC1, MUC2 proteins and antioxidant enzymes were increased by 37.2-195.8% (*p* = 0.001-0.012) in the CORT+MT group compared to the CORT group. Collectively, our data indicated that MT supplementation rescued the damage to the intestinal mucosa barrier, mitochondrial function, and the antioxidant capacity caused by CORT treatment.

### 3.4. Effect of MT Supplementation on CORT Treatment Enhances GR Synthesis and Transport and Triggers an Inflammation Response in the Colon

**Figure 2:**
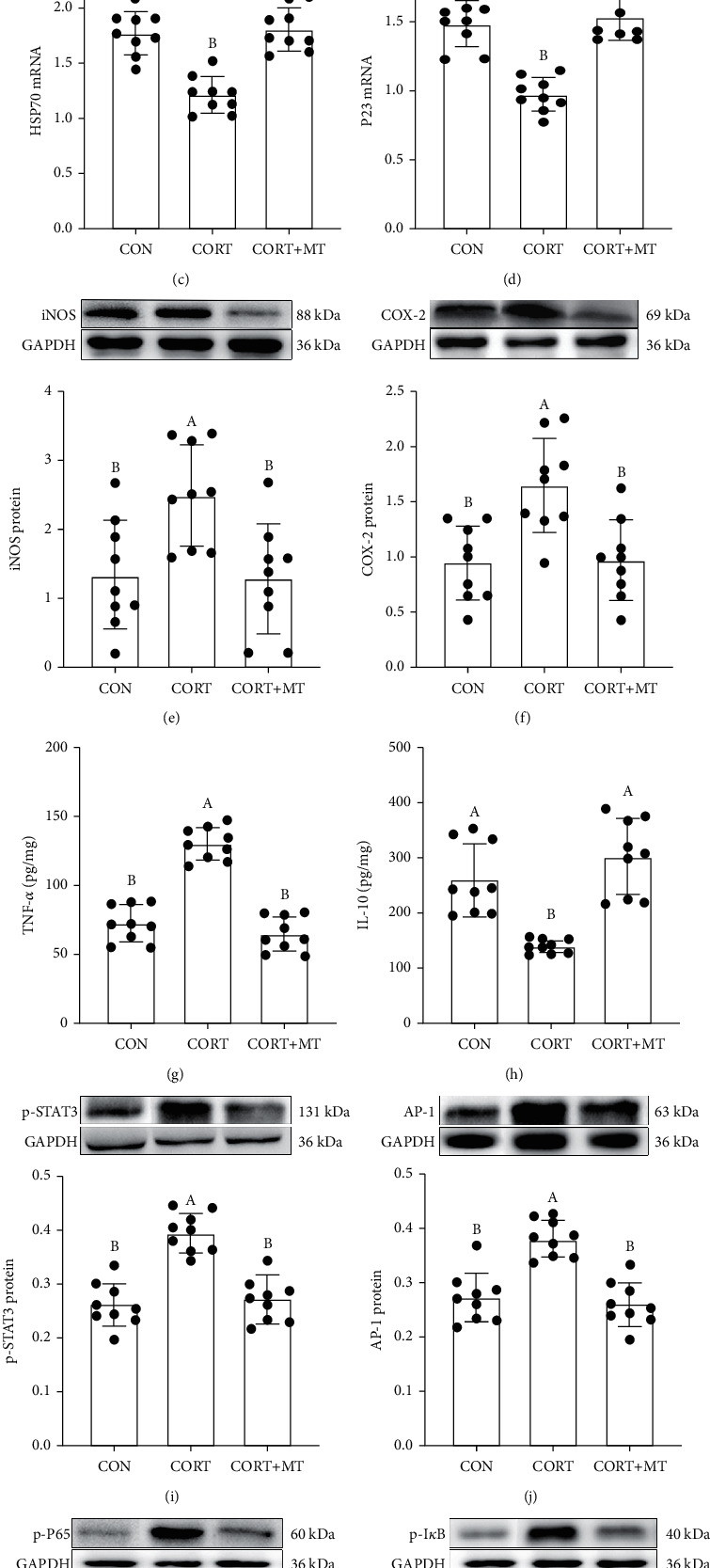
Effect of melatonin supplementation on improving CORT-induced GR synthesis and enhanced transport and inflammation response in mice. Colonic GR (a), HSP90 (b), HSP70 (c), P23 (d), iNOS (e), COX-2 (f), TNF-*α* (g), IL-10 (h), p-STAT3 (i), AP-1 (j), p-P65 (k), and p-I*κ*B (l) levels in the CON, CORT, and CORT+MT groups were measured by qRT-PCR, ELISA, and western blotting. Values are presented as the mean ± SE. Differences were assessed using ANOVA and are denoted as follows: different lowercase letters: *p* < 0.05; different uppercase letters: *p* < 0.01; and the same letter: *p* > 0.05.

We then examined whether CORT enhanced GR synthesis and transport and induced an inflammatory response in the colon (Figure 2). The western blotting results showed that expression of the GR protein and HSP90 mRNA was dramatically increased by 32.2% (*p* = 0.020, Figure 2(a)) and 87.8% (*p* ≤ 0.001, Figure 2(b)), while the HSP70 and P23 mRNA levels were decreased by 34.1% (*p* = 0.003, Figure 2(c)) and 38.4% (*p* ≤ 0.001, Figure 2(d)) in the CORT group relative to the CON group. However, MT supplementation obviously restored there changes, resulting in no statistically significant differences between the CON group and the MT-pretreated CORT group (*p* > 0.089).

Next, we assessed whether CORT treatment resulted in an inflammatory response. The results indicated that CORT caused an increase in the levels of colonic inflammatory molecules, such as iNOS, COX-2, and TNF-*α*, of 45.0% (*p* = 0.008, Figure 2(e)), 52.3% (*p* = 0.009, Figure 2(f)), and 123.2% (*p* ≤ 0.001, Figure 2(g)) and a reduction in IL-10 of 60.4% (*p* = 0.008, Figure 2(h)) compared with the CON group. However, the stimulatory effects of CORT on the inflammatory response were reversed in the colon by MT supplementation (*p* > 0.056).

Similar to its effect on the inflammatory response, CORT activated the STAT3/AP-1/NF-*κ*B pathway in the colon. The expression levels of the p-STAT3, AP-1, p-P65, and p-I*κ*B proteins in CORT-treated mice were increased by 39.2% (*p* = 0.014, Figure 2(i)), 52.7% (*p* = 0.001, Figure 2(j)), 66.8% (*p* = 0.003, Figure 2(k)), and 101.3% (*p* ≤ 0.001, Figure 2(l)), respectively, relative to the CON group. After MT supplementation, however, there was no statistically significant difference between the CON group and the MT-pretreated CORT group (*p* > 0.207).

Collectively, these results indicated that MT supplementation reversed CORT-induced colonic GR synthesis and transport and suppressed the STAT3/SP-1/NF-*κ*B pathway activation-mediated inflammatory response.

### 3.5. Effect of MT Supplementation on CORT Treatment Alters the Gut Microbiota Composition of the Colon

**Figure 3:**
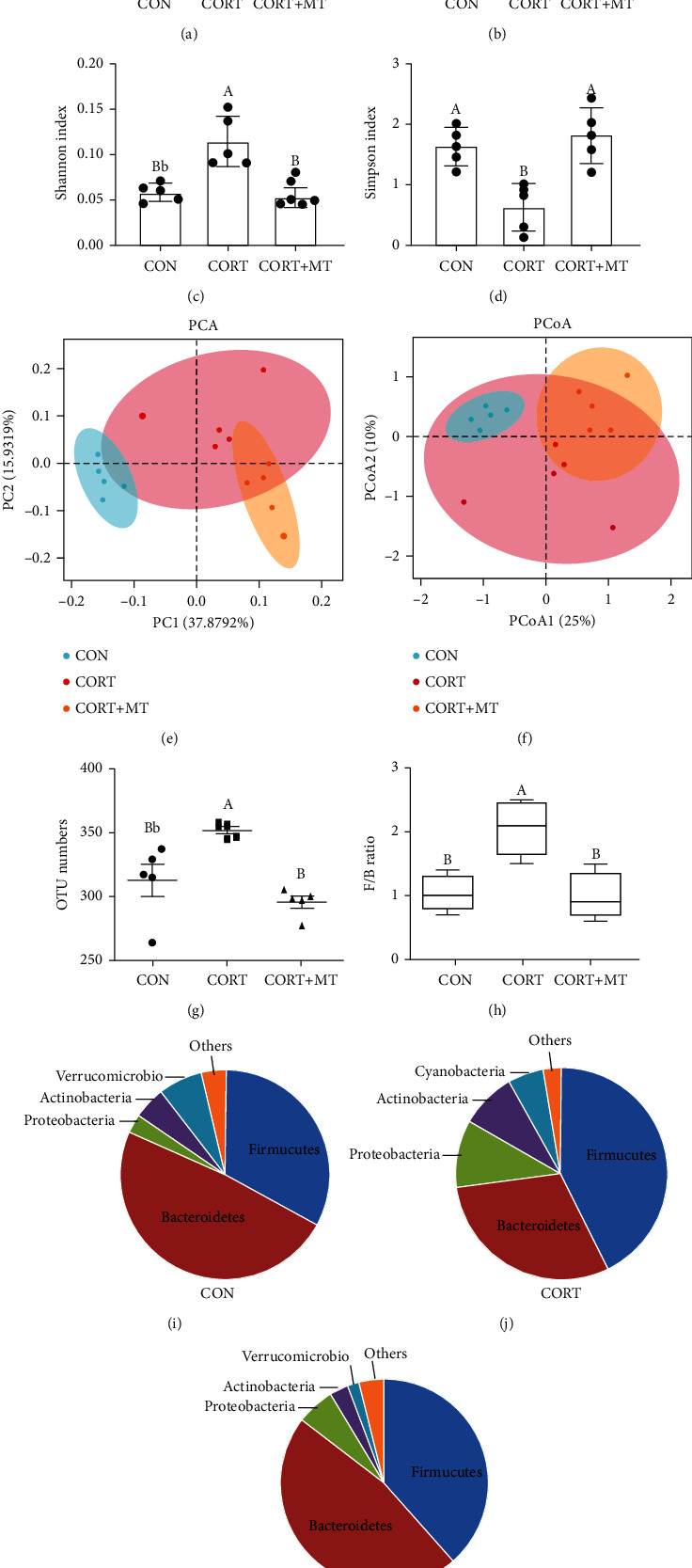
Effect of melatonin supplementation on improving CORT-induced colonic gut microbiota composition dysbiosis. The *α*-diversity includes diversity and richness. ACE index (a), Chao1 index (b), Shannon index (c), and Simpson index (d). The *β*-diversity shows the dispersion of each sample. Principal component analysis (PCA) (e) and PCoA score plot (f) score plot based on the Bray-Curtis score plot based on the OTU in the colon; OTU number (g); F : B ratio (h) and relative contribution of the top 4 phyla (I-K) in the CON (i), CORT (j), and CORT+MT (k) groups. Values are presented as the mean ± SE. Differences were assessed using ANOVA and are denoted as follows: different lowercase letters: *p* < 0.05; different uppercase letters: *p* < 0.01; and the same letter: *p* > 0.05.

We further investigated whether CORT and MT supplementation could affect the composition of the gut microbiota. Quantitative perspective analysis suggested that the ACE, Chao, and Shannon indexes were significantly increased by 51.5% (*p* = 0.001, Figure 3(a)), 69.9% (*p* = 0.008, Figure 3(b)), and 17.5% (*p* = 0.043, Figure 3(c)), respectively, while the Simpson index was markedly decreased by 37.5% (*p* = 0.032, Figure 3(d)). The *β*-diversity analysis indicated that the intestinal microbiota dispersion of CORT mice increased significantly relative to that of the CON group (Figures 3(e) and 3(f)). Moreover, there was an increase in the OTU numbers (26.9%, *p* = 0.012, Figure 3(g)) and the ratio of *Firmicutes* : *Bacteroidetes* (F : B, 31.2%, *p* = 0.003, Figure 3(h)) in the CORT group compared with the CON group. There was also a statistically significant increase in the relative abundance of Firmicutes (35.2%) and Proteobacteria (41.2%) and a decrease in the abundance of Bacteroidetes (43.9%) in CORT mice relative to control mice (Figures 3(i) and 3(j)). However, after supplementation of CORT mice with MT, the trends of all the indexes were restored to the levels of the CON group (Figures 3(k)), and there was no statistically significant difference between the CON group and the MT-supplemented group (*p* > 0.613). These results demonstrated that CORT upregulated the diversity, richness, and OTU numbers of colonic microbiota as well as the F : B ratio and that this microbial composition was significantly altered by MT supplementation.

**Figure 4:**
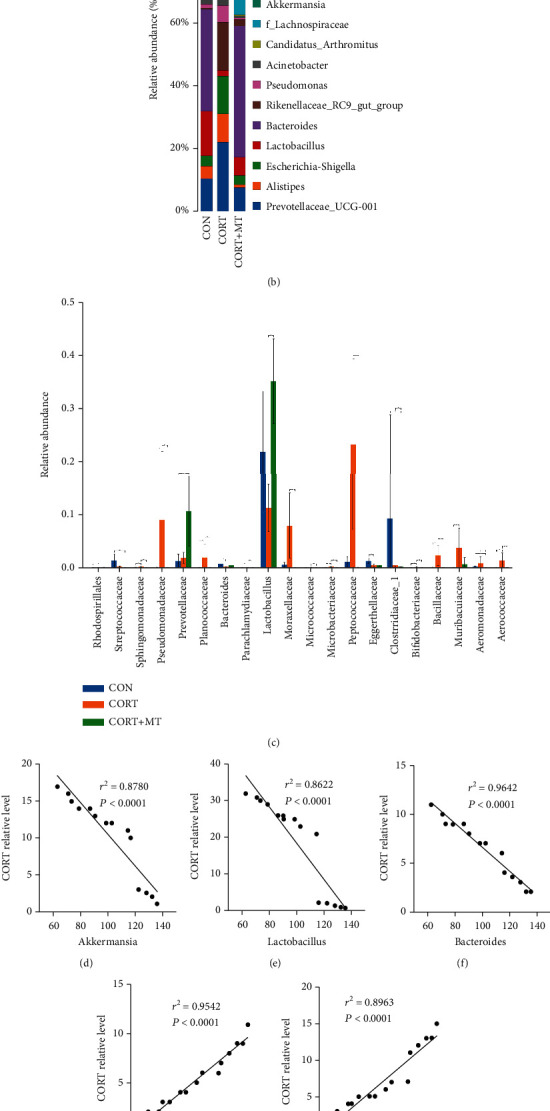
Effect of melatonin supplementation on improving CORT-induced colonic gut microbiota composition altered. Taxonomic cladogram obtained from LEfSe sequence analysis (a) in the colon. Biomarker taxa are highlighted by coloured circles and shaded areas. The diameter of each circle reflects the abundance of that taxa in the community; relative abundance of the top 15 genera (b) and the statistics of most change intestinal microbiota (c); correlation between the CORT level and the relative abundance of Akkermansia (d), Lactobacillus (e), Bacteroides (f), Prevotella (g), Allobaculum (h), Muribaculaceae (i), Proteobacteriaceae (j), and Rikenellaceae (k) in the CON, CORT, and CORT+MT groups in the colon. Values are presented as the mean ± SE. Differences were assessed using ANOVA and are denoted as follows: different lowercase letters: *p* < 0.05; different uppercase letters: *p* < 0.01; and the same letter: *p* > 0.05.

A cladogram representative of the colonic microbiota structure in mice further displayed the predominant bacteria and the greatest differences in taxa among the 3 treatment groups (Figure 4(a)). The results indicated that the predominant bacteria in the colons of CORT mice were *Bacillus*, *Prevotella*, *Alloprevotella*, *Pseudomonadaceae*, *Escherichia-Shigella*, and *Muribaculaceae*, while the predominant bacteria in the colons of CORT+MT mice were *Akkermansia*, *Bacteroidales*, and *Lactobacillus*, which are beneficial bacteria. Moreover, a genus level analysis demonstrated that CORT significantly decreased the relative abundances of *Lactobacillus*, *Bacteroides* and *Akkermansiaceae* and increased the relative abundances of *Prevotellaceae-UCG-001*, *Alistipes*, *Escherichia-Shigella*, *Rikenellaceae* and *Proteobacteriaceae*, while supplementation with MT restored the abundances of these bacteria to those of the CON group (Figure 4(b)).  Figure 4(c) shows that the relative abundances of *Pseudomonadaceae*, *Moraxellaceae*, *Bacillaceae*, *Aeromonadaceae*, *Aerococcaceae*, *Peptococcaceae*, and *Muribaculaceae* in the colons of the CORT group were significantly increased by 23.7% (*p* ≤ 0.001), 42.8% (*p* ≤ 0.001), 29.4% (*p* = 0.012), 63.1% (*p* = 0.010), 54.3% (*p* = 0.001), 54.1% (*p* = 0.001), and 48.1% (*p* = 0.002), respectively, relative to the CON group, while the levels of *Bacteroides* and *Lactobacillus* were significantly decreased by 53.1% (*p* ≤ 0.001) and 45.8% (*p* = 0.003), respectively, relative to the CON group. However, these changes were reversed by MT supplementation, and there was no significant difference among the CON, CORT, and CORT+MT groups (*p* > 0.052). These results demonstrated that MT supplementation suppressed the CORT-induced changes in the composition of the intestinal microbiota.

Furthermore, Pearson's correlation analysis showed a negative correlation between the relative abundances of *Akkermansia*, *Lactobacillus*, and *Bacteroides* and the plasma CORT level (*r*^2^ = 0.8780 and *p* < 0.0001, Figure 4(d); *r*^2^ = 0.8622 and *p* < 0.0001, Figure 4(e); and *r*^2^ = 0.9642 and *p* < 0.0001, Figure 4*r*^2^), as well as a positive correlation between the relative abundances of *Prevotella*, *Allobaculum*, *Muribaculaceae*, *Proteobacteriaceae* and *Rikenellaceae* and the plasma CORT level (*r*^2^ = 0.9542 and *p* < 0.0001, Figure 3(g); *r*^2^ = 0.8963 and *p* < 0.0001, Figure 4(h); *r*^2^ = 0.9111 and *p* < 0.0001, Figure 4(i); *r*^2^ = 0.8493 and *p* < 0.0001, Figure 4(j); and *r*^2^ = 0.8089 and *p* < 0.0001, Figure 4(k)). In general, MT supplementation significantly increased probiotics, which were negatively correlated with the CORT level, and suppressed pathogenic bacteria, which were positively correlated with the CORT level.

### 3.6. Effect of MT Supplementation on CORT Treatment Alters the Composition of Gut Microbiota Metabolites in the Colon

**Figure 5:**
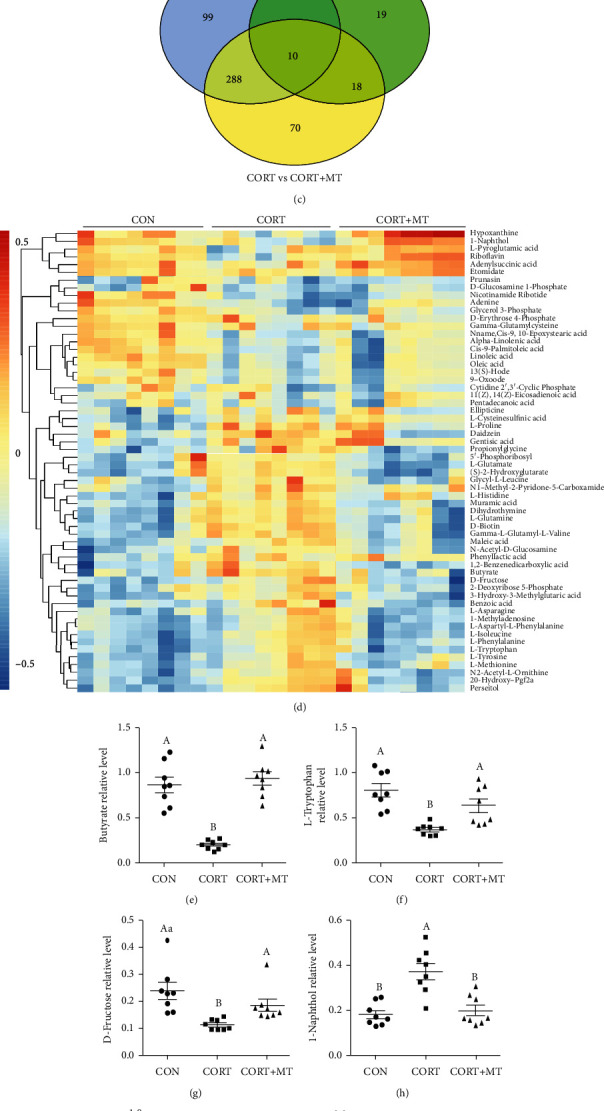
Effect of melatonin supplementation on improving CORT-induced colonic gut microbiota metabolite composition altered. The *β*-diversity of principal component analysis (PCA) (a), PCoA (b) and Venn (c) based on the microbiota metabolites in the CON, CORT, and CORT+MT groups; heat map (d) showing the relative abundance of the key identified 60 metabolites that were significantly altered by MT in CORT mice. The relative abundance of butyrate (e), L-tryptophan (f), D-fructose (g), 1-naphthol (h), hypoxanthine (i), and adenine (j) in the CON, CORT, and CORT+MT groups in the colon microbiota based on the heat map results. Values are presented as the mean ± SE. Differences were assessed using ANOVA and are denoted as follows: different lowercase letters: *p* < 0.05; different uppercase letters: *p* < 0.01; and the same letter: *p* > 0.05.

We further analyzed how the composition of gut microbiota metabolites responded to CORT treatment and MT supplementation. Our results suggested that CORT treatment led to a significant increase in colonic microbiota metabolite dispersion, which suggested a decrease in microbiota metabolite homogeneity (Figures 5(a) and 5(b)). The Venn diagram indicated that compared with the CON group, the levels of 412 metabolites were changed in the CORT group, while MT supplementation restored the levels of 298 of these metabolites (Figure 5(c)). Furthermore, we screened the 60 most changed metabolites in the three treatment groups (Figure 5(d)). Of these, the levels of 22 metabolites were significantly reduced and those of 38 metabolites were increased in the CORT group relative to the CON group. Specifically, there was a significant decrease in the contents of butyrate (68.9%, *p* ≤ 0.001, Figure 5(e)), L-tryptophan (58.4%, *p* = 0.001, Figure 5(f)) and D-fructose (48.3%, *p* ≤ 0.001, Figure 5(g)) as well as a significant increase in the contents of 1-naphthol (94.5%, *p* = 0.002, Figure 5(h)), hypoxanthine (89.3%, *p* = 0.002, Figure 5(i)) and adenine (100.2%, *p* = 0.008, Figure 5(j)) in the CORT group compared to the CON group. However, MT supplementation restored these levels to those in the CON group (*p* > 0.438). Collectively, these results indicated that MT supplementation reversed the CORT-induced alterations of colonic microbiota metabolites.

### 3.7. Effect of MT Supplementation on CORT Treatment Alters the Correlation between the Gut Microbiota and Microbiota Metabolites in the Colon

**Figure 6:**
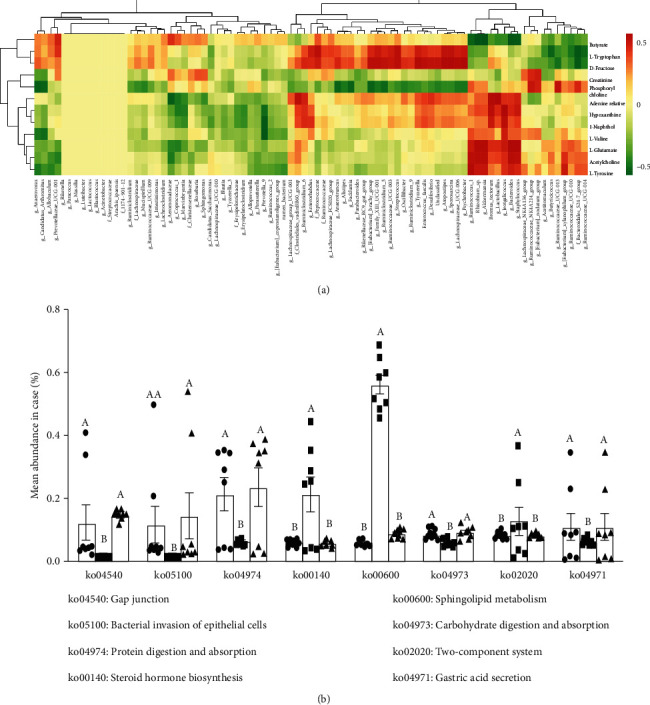
Correlation analysis between intestinal microbiota and metabolites in the colon of CORT mice with or without MT supplementation. Correlation of the most significant changes in intestinal microbiota and metabolites (a) and KEGG analysis (b) among the CON, CORT, and CORT+MT groups. Values are presented as the mean ± SE. Differences were assessed using ANOVA and are denoted as follows: different lowercase letters: *p* < 0.05; different uppercase letters: *p* < 0.01; and the same letter: *p* > 0.05.

We further analyzed the correlation between the gut microbiota and its metabolites in the CON, CORT, and CORT+MT groups. As shown in Figure 6(a), the most prominent alteration associated with the intestinal microbiota and its metabolites involved 12 metabolites and 83 intestinal microbiota. The levels of butyrate, L-tryptophan, and D-fructose were found to be directly proportional to the levels of *Butyricicoccus*, *Akkermansia*, and *Lactobacillus* but inversely proportional to the levels of *Alistipes*, *Peptostreptococcus*, and *Rikenellaceae*. The levels of adenine, hypoxanthine, and 1-naphthol were directly proportional to the levels of *Alistipes*, *Peptostreptococcus*, and *Rikenellaceae* and inversely proportional to the levels of *Butyricicoccus*, *Akkermansia*, and *Lactobacillus.* Further, KEGG analysis indicated CORT treatment mainly enhanced the steroid hormone biosynthesis, sphingolipid metabolism, and two-component system signaling pathways and inhibited the gap junction, bacterial invasion of epithelial cells, protein digestion and absorption, carbohydrate digestion, and absorption and gastric acid secretion pathways (Figure 6(b)). However, these changes were reversed by MT supplementation. It can be speculated that CORT treatment leads to changes in metabolite composition, especially decreases in the levels of butyrate and L-tryptophan, which in turn affect these pathways and functions, ultimately resulting in impaired intestinal barrier function.

### 3.8. FMT Promotes Reestablishment of the Intestinal Inflammation Response in Mice

As illustrated in Figures S3A and 3B, there was no significant difference of the plasma CORT and colonic ROS level among the F-CON, F-SD, F-SM, and F-R groups. Moreover, the expression level of p-STAT3, p-AP-1, p-I*κ*B, and p-P65 proteins increased by 64.3% (*p* = 0.006, Figure S3C), 63.9% (*p* ≤ 0.001, Figure S3D), 61.9% (*p* ≤ 0.001, Figure S3E), and 72.0% (*p* ≤ 0.001, Figure S3F) in the F-SD group, compared with the F-CON group. However, the stimulatory effects of F-SD on changes in colitis were reversed in the colon by F-SM supplementation.

Collectively, these results indicated that FMT from SD mice promoted reestablishment of the intestinal microecology to activate inflammation response, while MT supplementation suppressed this process.

### 3.9. Effect of MT Supplementation on CORT Treatment Triggers Oxidative Stress by Enhancing GR Synthesis and Transport in IECs

**Figure 7:**
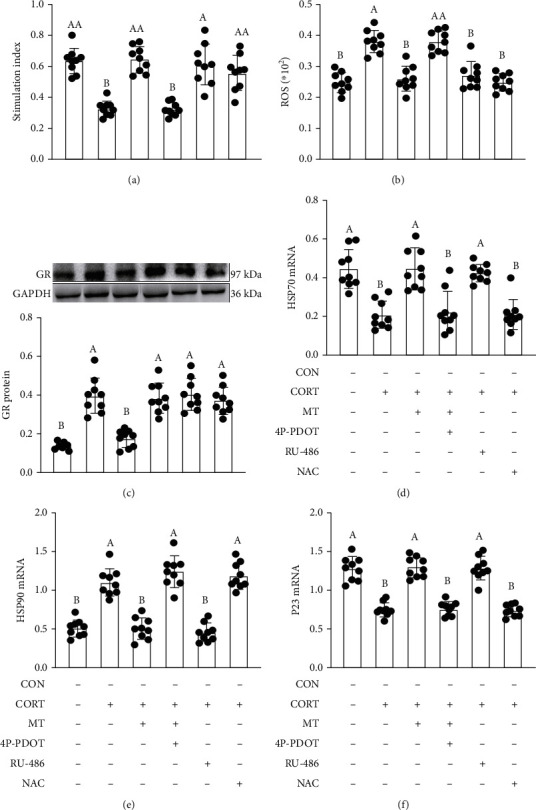
Improvement effect of melatonin on the relative levels of proliferation capacity (a), ROS content (b), GR (c), HSP70 (d), HSP90 (e), and P23 (f) in CORT-treated IECs. 4P-PDOT: an antagonist of MT2; RU-486: an antagonist of GR; NAC: a free radical scavenger. Values are presented as the mean ± SE. Differences were assessed using ANOVA and are denoted as follows: different lowercase letters: *p* < 0.05; different uppercase letters: *p* < 0.01; and the same letter: *p* > 0.05.

**Figure 8:**
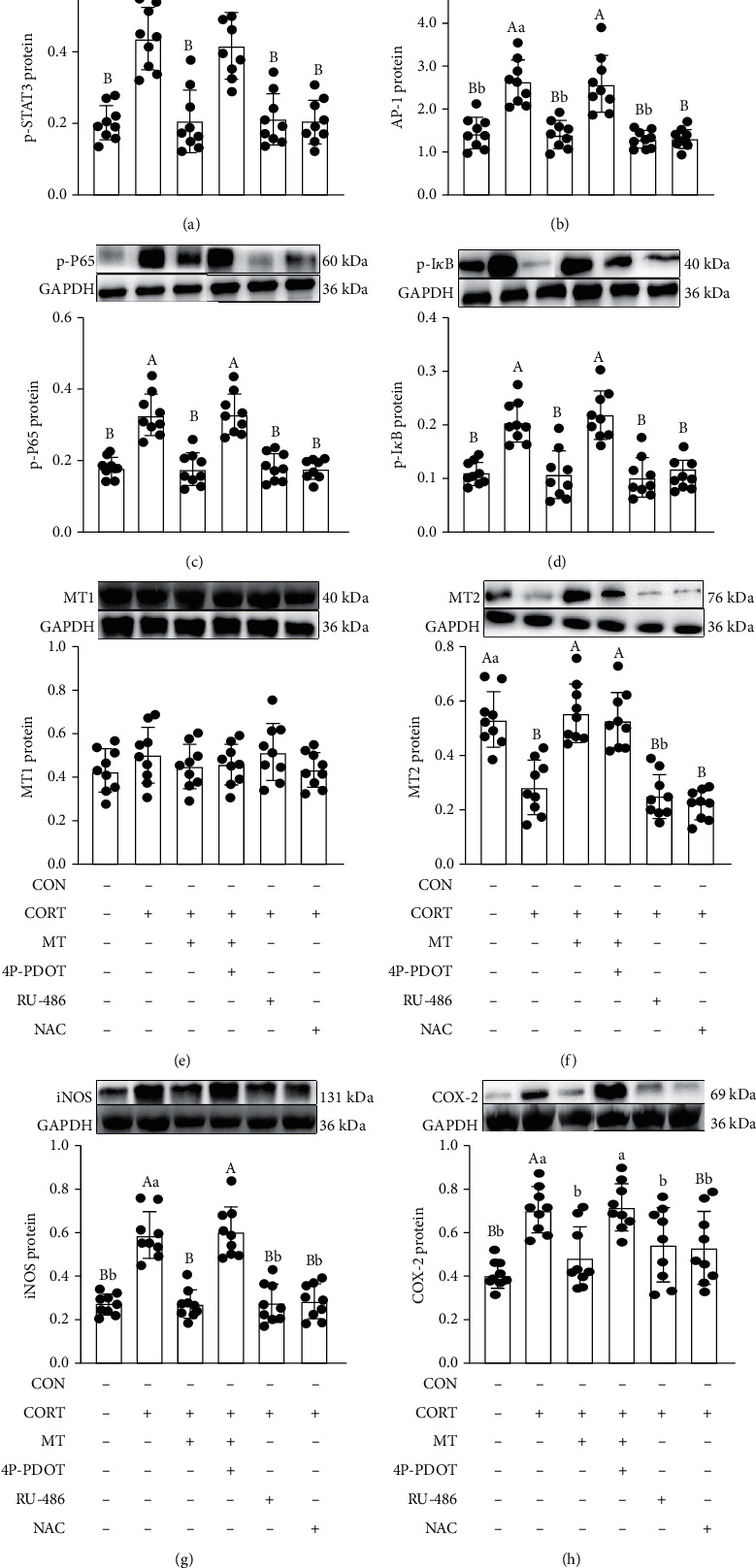
Improvement effect of melatonin on the relative levels of p-STAT3 (a), AP-1 (b), p-P65 (c), p-I*κ*B (d), MT1 (e), MT2 (f), iNOS (g), and COX-2 (h) in CORT-treated IECs. 4P-PDOT: an antagonist of MT; RU-486: an antagonist of GR; NAC: a free radical scavenger. Values are presented as the mean ± SE. Differences were assessed using ANOVA and are denoted as follows: different lowercase letters: *p* < 0.05; different uppercase letters: *p* < 0.01; and the same letter: *p* > 0.05.

We established a CORT-treated IECs model with or without MT administration (Figures 7 and 8) to investigate the mechanisms whereby MT supplementation reversed the CORT-induced intestinal homeostasis imbalance. CORT treatment induced an increase in the levels of ROS (52.7%, *p* = 0.010; Figure 7(b)), GR protein (168.1%, *p* = 0.002; Figure 7(c)) and HSP90 mRNA (178.8%, *p* = 0.005; Figure 7(e)) and a decrease in proliferative capacity (50.3%, *p* ≤ 0.001; Figure 7(a)) and the levels of HSP70 (53.4%, *p* = 0.029; Figure 7(d)) and P23 mRNA (43.2%, *p* = 0.003; Figure 7(f)) compared with the control IECs. However, these changes were dramatically suppressed by MT supplementation. The improvement effects of MT were replicated by pretreatment with RU-486, a GR antagonist. Pretreatment with NAC, a free radical scavenger, similarly imitated the effects of MT, increasing proliferative capacity (100.6%, *p* = 0.003) and decreasing the levels of ROS (34.5%, *p* = 0.010) in CORT+NAC-treated IECs relative to the CORT group; however, no changes were observed in the expression levels of GR proteins and HSP70, HSP90 and P23 mRNA (*p* > 0.050) after NAC treatment. Conversely, 4P-PDOT administration significantly suppressed the beneficial effects of MT and resulted in an increase in ROS (54.0%, *p* = 0.001), GR protein (67.8%, *p* ≤ 0.001), and HSP90 mRNA (61.2%, *p* = 0.004) levels and a decrease in proliferative capacity (74.5%, *p* = 0.003) and HSP70 (38.4%, *p* = 0.036) and P23 (79.0%, *p* ≤ 0.001) mRNA levels compared with vehicle IECs. Our results revealed that MT-mediated MT2 receptor ameliorated CORT-induced oxidative stress by suppressing GR synthesis and transport in the gut.

### 3.10. Effect of MT Supplementation on CORT Treatment Induces the Activation of STAT3/AP-1/NF-*κ*B Pathway via Inhibiting Oxidative Stress

Moreover, we observed an increase in the expression levels of p-STAT3 (97.7%, *p* = 0.031; Figure 8(a)), AP-1 (93.0%, *p* = 0.016; Figure 8(b)), p-P65 (96.1%, *p* = 0.010; Figure 8(c)), p-I*κ*B (109.1%, *p* = 0.022; Figure 8(d)), iNOS (113.9%, *p* = 0.018; Figure 8(g)) and COX-2 (69.2%, *p* = 0.016; Figure 8(h)) and a decrease in the expression level of MT2 (61.4%, *p* = 0.008; Figure 8(f)) in CORT-treated IECs compared with the vehicle group, while CORT treatment had no effect on the expression of MT1 protein (Figure 8(e)). However, MT pretreatment could suppress this process. After treatment with RU-486 (GR antagonist), which had similar effects to MT, we observed a decrease in expression of p-STAT3 (62.7%, *p* = 0.014), AP-1 (43.7%, *p* = 0.010), p-P65 (38.4%, *p* = 0.018), p-I*κ*B (51.1%, *p* = 0.017), iNOS (55.0%, *p* = 0.014), and COX-2 (42.8%, *p* = 0.001) in CORT+RU-486-treated IECs compared with the CORT-treated group, while RU-486 had no effect on MT1 and MT2 expression. Moreover, treatment with NAC, which inhibits oxidative stress, also restored the changes induced by CORT. Pretreatment with 4P-PDOT reversed the therapeutic effects of MT and failed to improve the changes induced by CORT. The expression of p-STAT3 (47.0%, *p* = 0.047), AP-1 (50.9%, *p* = 0.006), p-P65 (43.3%, *p* = 0.016), p-I*κ*B (56.3%, *p* = 0.015), iNOS (55.3%, *p* = 0.014) and COX-2 (41.7%, *p* = 0.015) was increased in CORT+MT+4P-PDOT-treated IECs relative to CORT+MT-treated IECs, indicating that MT-mediated MT2 ameliorated the CORT-induced inflammatory response, resulting from STAT3/AP-1/NF-*κ*B pathway activation.

## 4. Discussion

Our previous studies have demonstrated that SD, as a physiological stressor, induced an excessive CORT, gut microbiota disturbances, and colitis phenotype [2]. Moreover, the present study indicated the mice, receiving faeces microbiota from SD mice also suffered colitis phenotype and intestinal microbiota disorder, but no changes in plasma CORT were observed. However, melatonin supplementation reversed all inductions in SD-mice and transplanting faeces microbiota from SD+MT mice significantly restored SD-induced colitis and intestinal microbiota imbalance. These results emphasized the core role of overproduction CORT in SD-induced gut microbiota dysbiosis and revealed that MT-mediated gut microbiota homeostasis exerted a improvement effect in SD-induced colitis mice.

To verify the key role of excessive CORT in SD caused gut microbiota disorder, we established a CORT-treated mice. Results showed that CORT treatment induced a colitis phenotype. Meanwhile, microbial composition analysis in the present study revealed there was an increase in the diversity and richness, dispersion, OTU numbers, and the F : B ratio in CORT-treated mice. Recent studies have also shown that GCs may cause a disorder in gut microbes [11] and an increase in the F : B ratio, which is regarded as a key indicator of major changes in the gut microbiota. It has generally been assumed that GCs increase the risk of proliferation of pathogenic bacteria [19]. Our study also revealed a higher abundance of harmful bacteria (*Prevotella* and *Allobaculum)* and a lower abundance of beneficial bacteria (*Akkermansia*, *Bacteroides*, *Peptostreptococcus* and *Lactobacillus*) in the colons of mice exposed to CORT. *Bacteroides* and *Lactobacillus* are responsible for catabolizing butyrate and L-tryptophan, respectively [20]. Consistent with the microbial results, our metabolomics analysis further indicated that the relative abundance of butyrate and tryptophan in CORT mice was significantly reduced. The intestinal microbiota and its metabolites are integral mediators of intestinal barrier function and intestinal permeability and are capable of both perturbing and enhancing intestinal barrier integrity by modulating immune responses, oxidative stress, inflammation, vagal signaling, and nutrient availability [21]. Further, there was an increase in the expression levels of p-STAT3, AP-1, p-P65 and p-I*κ*B proteins and proinflammatory factors (COX-2, iNOS and TNF-*α*) in CORT mice. Research has revealed that butyrate promotes proinflammatory cytokine production (e.g., IL-6, IL-8, IL-1*β* and TNF-*α*) by enhancing NF-*κ*B activation in epithelial cells [22]. Moreover, in mice and piglets, the tryptophan supplementation reduced the symptoms of DSS-induced colitis, improved histology and intestinal permeability, and decreased the levels of local inflammatory mediators, such as IFN-*γ* and TNF-*α* [23]. Thus, our results revealed that SD-mediated CORT overproduction induced intestinal microbiota disorder and further triggered inflammation response, ultimately resulting in the occurrence of colitis.

We further investigated the cellular and molecular mechanisms underlying the cross-talk between the CORT overproduction and the gut microbiota. There was a reduction in the expression levels of HSP70 and P23 mRNA and an increase in GR protein and HSP90 mRNA in CORT-treated mice. The HSP90-GR-CORT complex, stabilized by cochaperones P23 and HSP70, ameliorated in CORT mice [24]; it moves to the nucleus and interacts with the glucocorticoid response element to regulate downstream signaling pathways [25]. This implies that GR synthesis and transport are enhanced and that the negative feedback regulation of the HPA axis is weakened in CORT mice. Consistent with HPA axis dysregulation, CORT treatment also destroyed intestinal mitochondrial function and antioxidant capacity in the colon. A high dose of GCs can indirectly induce oxidative stress through the depletion of antioxidant molecules or inhibition of antioxidant enzymes [26]. One possibility determined by our study is that dysregulation of mitochondrial functions induced the overproduction of ROS, creating a hypoxic niche due to oxygen consumption, and can affect the gut microbiota through perturbation of the normal intestinal environment, allowing bacterial antigens to penetrate the epithelium and stimulate an immune response [27]. Therefore, we concluded that the depletion of antioxidants, production of ROS, and lipid peroxidation after CORT exposure play an important role in intestinal microbiota imbalance-mediated inflammation response induced colitis.

Interestingly, our results showed that MT supplementation normalized the plasma CORT level in CORT-treated mice. Consistent with the reduction in CORT levels, MT enhanced the colonic mitochondrial function and antioxidant capacity and reversed intestinal microbiota metabolite dysbiosis and intestinal mucosa barrier damage. We further explored the specific mechanisms by which MT alleviated intestinal mucosa damage via in vitro experiments. Significant increases in the ROS content and expression level of the GR protein and HSP90 mRNA and a decrease in HSP70 and P23 mRNA expression were observed in colonic CORT-treated IECs. However, MT supplementation reversed the changes induced by CORT. Similarly, RU-486, a corticoid receptor antagonist replicated the effects of MT, inducing a downregulation of ROS and HSP90 mRNA expression and an upregulation of HSP70 and P23 mRNA expression, indicating that GCs have direct effects on GR transcription regulation-mediated oxidative stress. A previous study also demonstrated that an increase in GR levels related to HPA axis disorder has close interactions with oxidative stress [28]. To further explore the mechanisms involved in the inflammatory response induced by oxidative stress in CORT-treated IECs, we focused on the NF-*κ*B pathway, which is a critical mediator of oxidative stress. We also found NAC had a negative effect on the expression levels of p-STAT3, AP-1 p-P65, and p-I*κ*B proteins, but no changes in the GR synthesis and transport were observed, which suggested that oxidative stress alters the activities of intracellular effectors of STAT3/AP-1/NF-*κ*B [29]. Importantly, we observed that 4P-PDOT, an antagonist of MT2, counteracted the ameliorative effects of MT and promoted the expression level of GR protein and ROS and activated the STAT3/AP-1/NF-*κ*B pathway. Previous studies have also demonstrated that overexpression of MT2, but not MT1, had a significant amelioration effect on oxidative stress, endoplasmic reticulum stress, and mitochondrial dysfunction [30]. These results indicated that MT, mediated by the MT2 receptor, could inhibit CORT-induced GR synthesis and transport and activation of the STAT3/AP-1/NF-*κ*B pathway, further suppressing oxidative stress, which mediates the imbalance in the intestinal microbiota and its metabolites and, ultimately, leads to intestinal mucosa barrier dysfunction caused by SD.

This study suggested that the CORT, as a hazardous substance, plays a key role in SD-mediated gut microbiota dysbiosis-induced colitis. However, intraperitoneal injection of MT is an effective way to mitigate CORT-induced metabolic side effects via the MT2 receptor in SD mice. Importantly, our data provide novel evidence for the beneficial effects of MT as a physiological regulator of excessive CORT-mediated intestinal diseases and also support the recent broadening of the definition of probiotics to include MT-based strategies.

## 5. Conclusion

In conclusion, melatonin-mediated MT2 weakened GR feedback, suppressed oxidative stress, restored the intestinal microbiota and its metabolites homeostasis, and inactivated the STAT3/AP-1/NF-*κ*B pathway-induced inflammatory response, further improving SD-induced colitis.

## Figures and Tables

**Table 1:** Primers of target genes and reference gene.

Gene	Sense	Antisense
HSP90	ACGAGGAAGAGAAGAAGAAAATGG	GCAGGGTGAAGACACAAGCC
HSP70	CGGTGCCCGCCTACTTC	TCCTTCTTGTGCTTCCTCTTGA
P23	ATGCGTTTGGAGAAGGACAGA	CAGGGATGAAGTGATGGTGAGA
GAPDH	CCGAGAATGGGAAGCTTGTC	TTCTCGTGGTTCACACCCATC
Firmicutes	GGAGCATGTGGTTTAATTCGAAGCA	AGCTGACGACAACCATGCAC
Bacteroidetes	GAGAGGAAGGTCCCCCAC	CGCTACTTGGCTGGTTCAG
Proteobacteria	GGTTCTGAGAGGAGGTCCC	GCTGGCTCCCGTAGGAGT
Prevotellaceae	CACCAAGGCGACGATCA	GGATAACGCCCGGACCT
Escherichia coli	GGAGCAAACAGGATTAGATACCC	AACCCAACATTTCACAACACG

## Data Availability

All data generated or analyzed during this study are included in this published article.

## Supplementary Materials

Supplementary 1 Figure S1: Effects of melatonin on HPA axis activity and intestinal microbiota composition in sleep-deprived mice. ROS (A) level, GR (B) protein, HSP90 (C) mRNA, HSP70 (D) mRNA, and P23 (E) mRNA expression. Relative abundance of Bacteroidetes (F), Firmicutes (G), Proteobacterium (H) and Prevotellaceae (I) in the colon of the F-CON, F-SD, F-SM, and F-R groups. Values are presented as the mean ± SE. Differences were assessed using ANOVA and are denoted as follows: different lowercase letters: *p* < 0.05; different uppercase letters: *p* < 0.01; and the same letter: *p* > 0.05.

Supplementary 2 Figure S2: FMT reestablished the intestinal microecology similar to CON, SD and SD+MT mice. Immunohistochemical staining of MUC2 (A), ZO-1 (B), and Claudin-1 (C) in colon sections (scale: 50 *μ*m). IOD of MUC2 (D), ZO-1 (E), and Claudin-1 (F) proteins. DAI score (G); relative luciferase activity for colonic permeability (H); relative protein level of Card9 (I); IL-17 concentrations (J); relative abundance of Bacteroidetes (K), Firmicutes (L), Prevotellaceae (M), and Proteobacterium (N) in the colon of the F-CON, F-SD, F-SM, and F-R groups (*n* = 12). Values are presented as the mean ± SE. Differences were assessed using ANOVA and are denoted as follows: different lowercase letters: *p* < 0.05; different uppercase letters: *p* < 0.01; and the same letter: *p* > 0.05.

Supplementary 3 Figure S3: FMT reestablished the oxidative stress and inflammation response similar to CON, SD, and SD+MT mice. Serum CORT (A) concentrations were measured by ELISA (*n* = 8). ROS (B) level and expression level of p-STAT3 (C), p-AP-1 (D), p-I*κ*B (E), and p-P65 (F) proteins in the F-CON, F-SD, F-SM, and F-R groups were examined by western blotting, and relative protein levels were normalized to GAPDH. Values are presented as the mean ± SE. Differences were assessed using ANOVA and are denoted as follows: different lowercase letters: *p* < 0.05; different uppercase letters: *p* < 0.01; and the same letter: *p* > 0.05.
