# Supplementary figures and images for: Melatonin Ameliorates Corticosterone-Mediated Oxidative Stress-Induced Colitis in Sleep-Deprived Mice Involving Gut Microbiota
Source: Oxid Med Cell Longev. 2021 Jun 23;2021:9981480. doi: 10.1155/2021/9981480 (PMC8246302; doi:10.1155/2021/9981480)

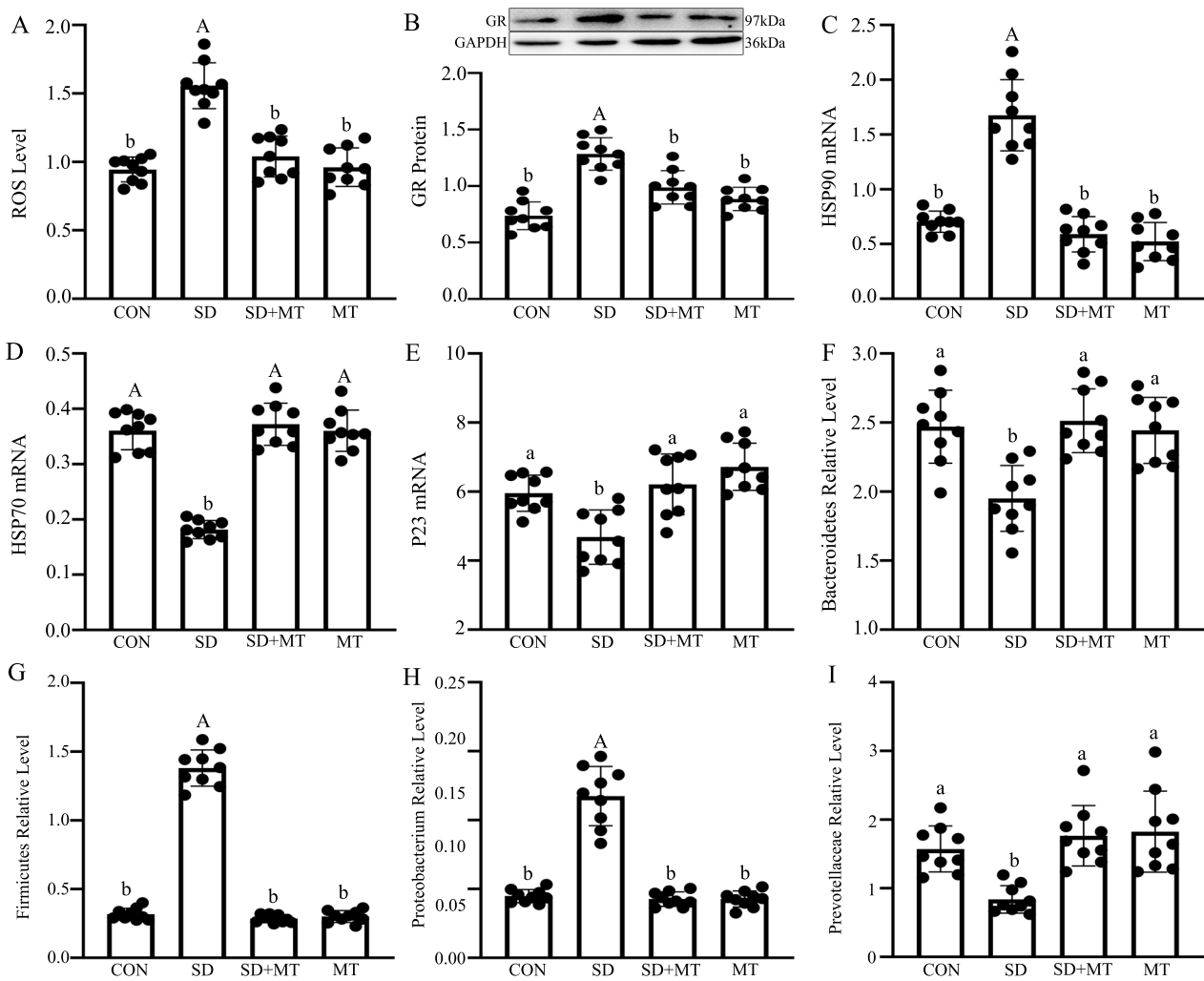

Supplement: Supplementary 1 — Figure S1: Effects of melatonin on HPA axis activity and intestinal microbiota composition in sleep-deprived mice. ROS (A) level, GR (B) protein, HSP90 (C) mRNA, HSP70 (D) mRNA, and P23 (E) mRNA expression. Relative abundance of Bacteroidetes (F), Firmicutes (G), Proteobacterium (H) and Prevotellaceae (I) in the colon of the F-CON, F-SD, F-SM, and F-R groups. Values are presented as the mean ± SE. Differences were assessed using ANOVA and are denoted as follows: different lowercase letters: p < 0.05; different uppercase letters: p < 0.01; and the same letter: p > 0.05. [file 9981480.f1.pdf]

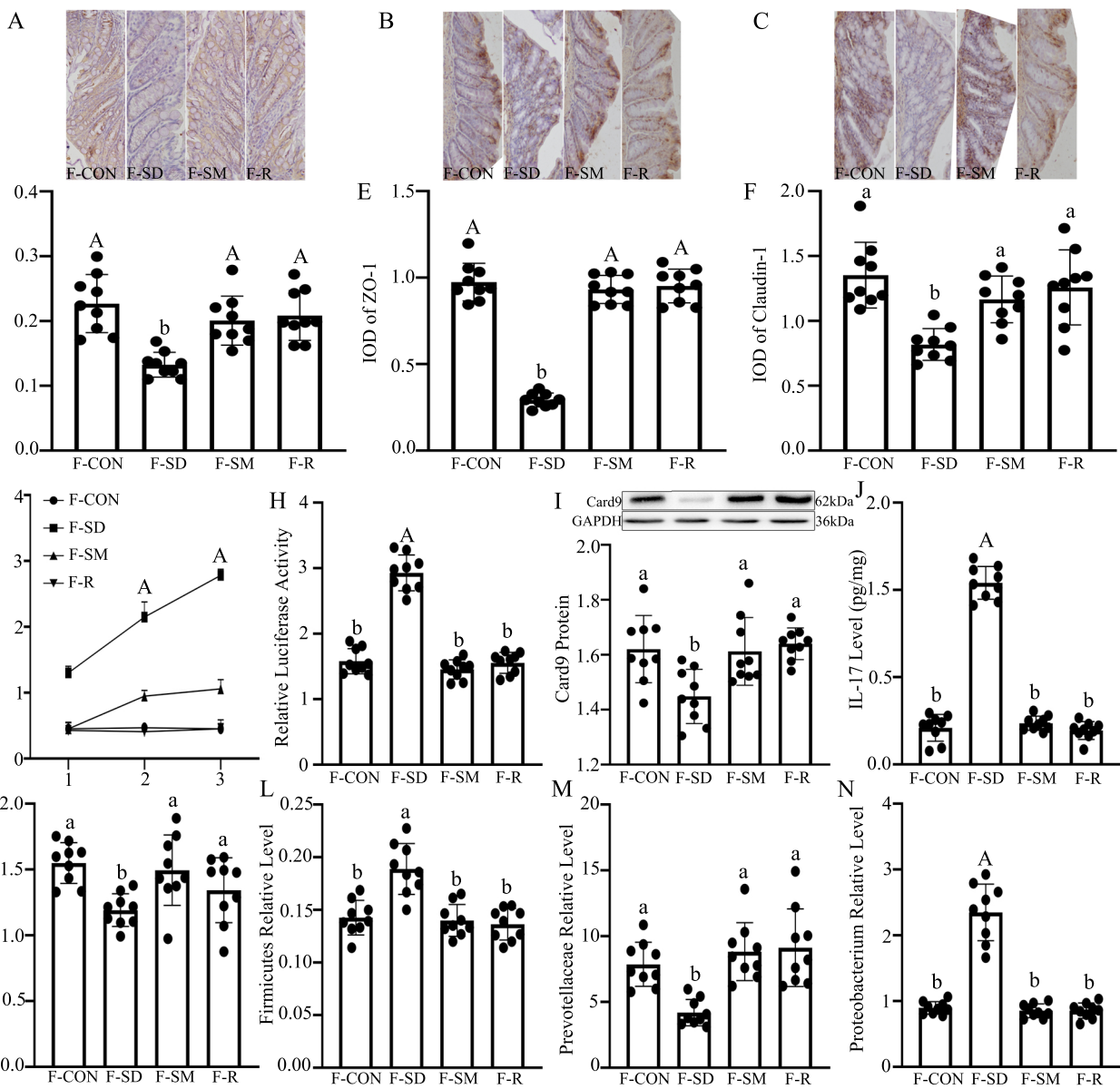

Supplement: Supplementary 2 — Figure S2: FMT reestablished the intestinal microecology similar to CON, SD and SD+MT mice. Immunohistochemical staining of MUC2 (A), ZO-1 (B), and Claudin-1 (C) in colon sections (scale: 50 μm). IOD of MUC2 (D), ZO-1 (E), and Claudin-1 (F) proteins. DAI score (G); relative luciferase activity for colonic permeability (H); relative protein level of Card9 (I); IL-17 concentrations (J); relative abundance of Bacteroidetes (K), Firmicutes (L), Prevotellaceae (M), and Proteobacterium (N) in the colon of the F-CON, F-SD, F-SM, and F-R groups (n = 12). Values are presented as the mean ± SE. Differences were assessed using ANOVA and are denoted as follows: different lowercase letters: p < 0.05; different uppercase letters: p < 0.01; and the same letter: p > 0.05. [file 9981480.f2.pdf]

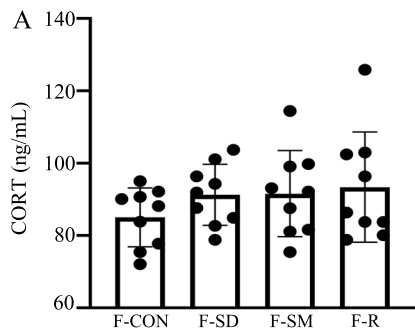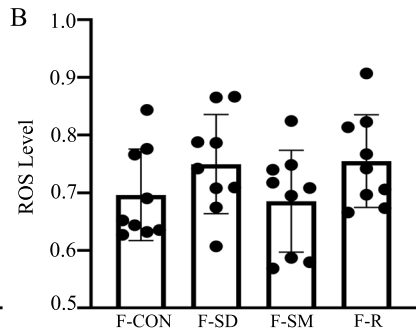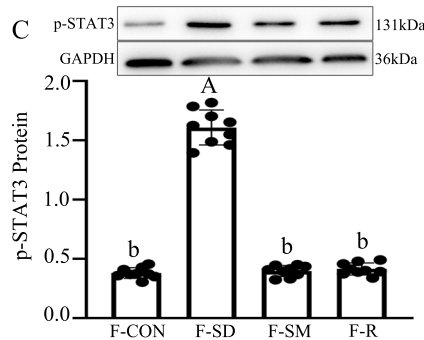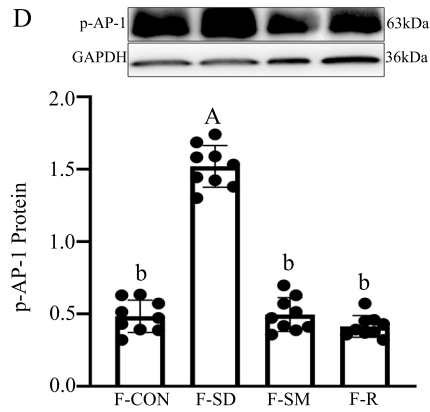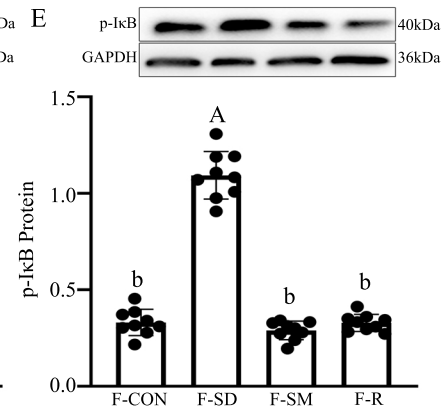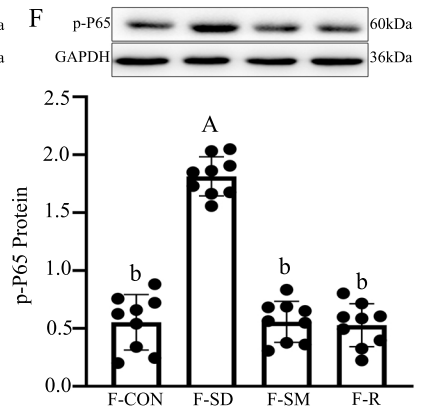

Supplement: Supplementary 3 — Figure S3: FMT reestablished the oxidative stress and inflammation response similar to CON, SD, and SD+MT mice. Serum CORT (A) concentrations were measured by ELISA (n = 8). ROS (B) level and expression level of p-STAT3 (C), p-AP-1 (D), p-IκB (E), and p-P65 (F) proteins in the F-CON, F-SD, F-SM, and F-R groups were examined by western blotting, and relative protein levels were normalized to GAPDH. Values are presented as the mean ± SE. Differences were assessed using ANOVA and are denoted as follows: different lowercase letters: p < 0.05; different uppercase letters: p < 0.01; and the same letter: p > 0.05. [file 9981480.f3.pdf]
